# Increased Wnt5a in squamous cell lung carcinoma inhibits endothelial cell motility

**DOI:** 10.1186/s12885-016-2943-4

**Published:** 2016-11-23

**Authors:** J. Rapp, E. Kiss, M. Meggyes, E. Szabo-Meleg, D. Feller, G. Smuk, T. Laszlo, V. Sarosi, T. F. Molnar, K. Kvell, J. E. Pongracz

**Affiliations:** 1 Department of Pharmaceutical Biotechnology, School of Pharmacy, University of Pecs, 2 Rokus Str, Pecs, 7624 Hungary; 2 Medical Microbiology and Immunity, University of Pécs, 12 Szigeti Str, Pécs, 7624 Hungary; 3 Biophysics, University of Pécs, 12 Szigeti Str, Pécs, 7624 Hungary; 4 Pathology, University of Pécs, 12 Szigeti Str, Pécs, 7624 Hungary; 5 Internal Medicine, Pulmonology, University of Pécs, 2 Rakoczi Str, Pécs, 7623 Hungary; 6 Operational Medicine, University of Pécs, 12 Szigeti Str, Pécs, 7624 Hungary; 7 János Szentágothai Research Centre, University of Pécs, 20 Ifjúság Str, Pecs, 7622 Hungary; 8 Department of Surgery, Thoracic Surgery Unit, Petz A Hospital, 2-4 Vasvari Str, Győr, 9023 Hungary; 9 Humeltis Ltd, János Szentágothai Research Center, University of Pécs, 20 Ifjúság Str, Pécs, 7622 Hungary

**Keywords:** Lung cancer, NSCLC, Angiogenesis, Wnt, PPARgamma

## Abstract

**Background:**

Angiogenesis is important both in normal tissue function and disease and represents a key target in lung cancer (LC) therapy. Unfortunately, the two main subtypes of non-small-cell lung cancers (NSCLC) namely, adenocarcinoma (AC) and squamous cell carcinoma (SCC) respond differently to anti-angiogenic e.g. anti-vascular endothelial growth factor (VEGF)-A treatment with life-threatening side effects, often pulmonary hemorrhage in SCC. The mechanisms behind such adverse reactions are still largely unknown, although peroxisome proliferator activator receptor (PPAR) gamma as well as Wnt-s have been named as molecular regulators of the process. As the Wnt microenvironments in NSCLC subtypes are drastically different, we hypothesized that the particularly high levels of non-canonical Wnt5a in SCC might be responsible for alterations in blood vessel growth and result in serious adverse reactions.

**Methods:**

PPARgamma, VEGF-A, Wnt5a, miR-27b and miR-200b levels were determined in resected adenocarcinoma and squamous cell carcinoma samples by qRT-PCR and TaqMan microRNA assay. The role of PPARgamma in VEGF-A expression, and the role of Wnts in overall regulation was investigated using PPARgamma knock-out mice, cancer cell lines and fully human, in vitro 3 dimensional (3D), distal lung tissue aggregates. PPARgamma mRNA and protein levels were tested by qRT-PCR and immunohistochemistry, respectively. PPARgamma activity was measured by a PPRE reporter system. The tissue engineered lung tissues expressing basal level and lentivirally delivered VEGF-A were treated with recombinant Wnts, chemical Wnt pathway modifiers, and were subjected to PPARgamma agonist and antagonist treatment.

**Results:**

PPARgamma down-regulation and VEGF-A up-regulation are characteristic to both AC and SCC. Increased VEGF-A levels are under direct control of PPARgamma. PPARgamma levels and activity, however, are under Wnt control. Imbalance of both canonical (in AC) and non-canonical (in SCC) Wnts leads to PPARgamma down-regulation. While canonical Wnts down-regulate PPARgamma directly, non-canonical Wnt5a increases miR27b that is known regulator of PPARgamma.

**Conclusion:**

During carcinogenesis the Wnt microenvironment alters, which can downregulate PPARgamma leading to increased VEGF-A expression. Differences in the Wnt microenvironment in AC and SCC of NSCLC lead to PPARgamma decrease via mechanisms that differentially alter endothelial cell motility and branching which in turn can influence therapeutic response.

**Electronic supplementary material:**

The online version of this article (doi:10.1186/s12885-016-2943-4) contains supplementary material, which is available to authorized users.

## Background

Lung cancer (LC) with disappointing survival statistics is a leading cause of morbidity in both genders worldwide [1, 2]. The two main types of LC-s are small cell lung cancer (SCLC) and non-small cell lung cancer (NSCLC) where the latter can be further classified into adeno- (AC), squamous cell- (SCC), large cell (LCC) and various mixed types carcinomas accounting all together for approximately 85% of all LC cases [3]. As the majority of patients are diagnosed at an advanced stage of the disease, the outcome is poor and the overall 5-year survival rarely exceeds 15% [4]. Naturally, earlier recognition would improve the outcome, but currently only a few treatment options are available to lung cancer sufferers [5] and the more specific treatments are largely based on identified driver mutations [6]. Unfortunately, only a small percentage of NSCLC patients have such characteristic mutations therefore the majority cannot benefit from targeted therapy [7]. Recognition that new blood vessel formation is important to tumor growth lead to development of angiogenesis inhibitors [8] to block tumor growth and disease progression. The first monoclonal antibody –bevacizumab- was approved against the human vascular endothelial growth factor A (VEGF-A) a key regulator of angiogenesis [9]. As VEGF-A promotes endothelial cell survival, migration, proliferation and vascular permeability it appeared an ideal target to “starve” the tumor and lead to tumor regression. Although VEGF-A is the main signaling molecule in pathological angiogenesis and is upregulated in many tumors [10] including in NSCLC-s [11] success of anti-angiogenic therapy in human cancers remained far from impressive.

Despite some positive results using bevacizumab mostly in combination therapy [12], patients mainly with squamous histology were excluded from treatment as increased risk of fatal side effects were observed [13]. The reasons for limited responsiveness or increased hemorrhage are still unknown, but several ideas have come to light. For example, the two types of NSCLC-s not only differ in genomic mutations [14], but AC and SCC also possess different intra-tumoral blood vessel formations. Kojima et al. have reported that microvessel density is higher in AC than SCC [15], while Yazdani et al. have hypothesized that intratumoral vessels are less covered by pericytes in SCC than AC, leading to more vulnerable and fragile vascular wall with increased necrosis in newly formed vessels in SCC [16]. As alternative signaling pathways, such as basic fibroblast growth factor (bFGF), platelet derived growth factor (PDGF) as well as miRNAs, especially the pro-angiogenic miR-27b and the miR-200 family [17, 18] also play a significant role in the regulation of angiogenesis; solely blocking VEGF-A simply cannot provide a therapeutic solution in NSCLCs [19]. Additionally, the underlying signaling mechanisms have not been fully elucidated that would also be essential to stratify the patient population subjected to anti-angiogenic therapies.

One of the controversial regulators of VEGF-A is peroxisome proliferator-activated receptor gamma (PPARgamma) that has been reported to inhibit endothelial cell function [20] and vasodilatation [21]. According to the growing literature, PPARgamma can either activate or inhibit VEGF-A mediated endothelial cell response [22] depending on the modulatory effect of the surrounding molecular microenvironment [23]. Investigation of molecular interactions revealed that PPARgamma expression in the presence of PDGF results in good prognosis, whereas bFGF diminishes the positive role of PPARgamma in tumor recurrence [24]. PPARgamma regulates endoglin (CD105) [25] that is a VEGF-A induced endothelial cell proliferation marker [26] but is also responsible for the vascular tone [27]. As PPARgamma regulates nitric oxide synthase activity [28] it is also an important protein in vasorelaxation [29].

Interestingly, both PPARgamma [30, 31] and VEGF-A have been reported to be under Wnt control [32]. The Wnt family of secreted glyco-lipo-proteins control a wide variety of cellular processes including cell fate specification, cell proliferation, cell polarity and cell migration and are therefore important in both fetal development and carcinogenesis [33, 34]. Depending on the initial trigger one of the three main Wnt pathways are activated. Two non-canonical pathways including the Ca^2+^ and the planar cell polarity (PCP) pathways or the beta-catenin dependent canonical pathway [35]. Activation or mutation of molecules regulating Wnt signaling have been reported in many cancer types although mutations in NSCLC-s are rare [36]. As canonical and non-canonical Wnt pathways are differentially active in AC and SCC [37], we considered the possibility that differences in the Wnt microenvironment may be partly responsible for variations in the tumor angiogenic processes and therapeutic response. Especially, as Wnt-s, particularly the non-canonical Wnt5a, also regulate endothelial cell division, survival and migration [38] strengthening the hypothesis that angiogenesis is under Wnt control [32].

In the present study, we focused our attention on Wnt5a and PPARgamma to have a better insight into the regulation of angiogenesis in AC and SCC. Wnt5a was specifically selected as its up-regulation is characteristic to SCC tumors distinguishing the tumor microenvironment of SCC from AC [37]. We theorized that upregulation of non-canonical Wnt-s in SCC might also be responsible for alterations in blood vessel formation leading to more severe side effects to anti-VEGF therapies.

## Methods

### Ethical statement

All collected samples were treated anonymously. All patients were diagnosed with NSCLC, 23 of adenocarcinoma and 16 of squamous cell carcinoma. Patient characteristics are shown in Additional file 1: Table S1.

### Animals

Lungs were used from wild-type, PPARgamma+/− heterozygous and PPARgamma−/− KO mice of C57BL/6 J genetic background. The design to generate viable PPARgamma null mice was described previously [39]. Briefly, PPARgamma+/− / Sox2Cre + male mice were crossed with PPARgammafl/fl female mice to generate heterozygous PPARgammafl/- / Sox2Cre- and homozygous PPAR gammaΔfl/- / Sox2Cre + mice, wherein the floxed allele was recombined resulting in a null allele. Genotypes were determined by PCR using primers for Cre transgene, PPARgamma upstream loxP site and for the null allele. Mice were housed under minimal disease (MD) conditions. PPARgamma KO or heterozygous mice and their controls were kept in the Laboratory Animal Core Facility of the University of Debrecen that is registered to breed genetically-modified mouse strains (reg. no.: TMF/82-10/2015). 3–3 animals from the control and the PPARgamma KO group were sacrificed at the age of 3.5 months.

### Cell lines and primary cells

Human hTERT-immortalized primary human foreskin fibroblast cell line (F11, System bioscience Mountain View, CA, USA) and human lung adenocarcinoma A549 (American Type Culture Collection, Rockville, MD) cell line were used for the experiments. VEGF-A overexpressing F11 cell line was generated in our laboratory using lentiviral transfection (see below). Normal primary human small airway epithelial cells (SAEC), normal human lung fibroblast (NHLF) and human microvascular lung endothelial cells (HMVEC-L) were purchased from Lonza (Basel, Switzerland), isolated from anonymous donors of different ages and sex. F11 cells were used in experiments between passage numbers 2 to 5 and the cells were negative for cytokeratin. A549 cells were used between passage number 8 to 10 and epithelial characteristics were proved by cytokeratin positivity. All the cell cultures were regularly tested for mycoplasma infection [40].

### Cell cultures

F11 and normal human lung fibroblast (NHLF) cells were cultured at 37 °C, 5% CO_2_ in Fibroblast Growth Medium-2 (FGM-2) (Lonza, Basel, Switzerland). Small airway epithelial cell (SAEC) and human lung microvascular endothelial cell (HMVEC-L) cells were maintained at the same condition using small airway growth medium (SAGM) and endothelial growth medium (EGM-2 MV), according to the manufacturer’s recommendation (Lonza, Basel, Switzerland).

### Materials

Beta-catenin activator LiCl was obtained from Sigma-Aldrich (St. Louis, Missouri, USA) and used in 10 mM concentration [41]. Purified, recombinant human Wnt5a and Wnt11 (Chinese Hamster Ovary Cell Line, CHO-derived Gln38-Lys380) protein was purchased from R&D Systems (Minneapolis, USA) and used at a concentration of 1 μg/ml [37]. PPARgamma agonist rosiglitazone (RSG) and antagonist GW9662 were obtained from Sigma-Aldrich (St. Louis, USA) and used at 10 μM concentrations each [42, 43]. During the experiments, A549 and F11 cells were treated with 10 mM LiCl (Sigma-Aldrich, St. Louis, Missouri, USA), 10 μM RSG and 10 μM GW9662 (Sigma-Aldrich, St. Louis, Missouri, USA) for 48 h.

### Three dimensional lung tissues [44]

To create a fully human 3D lung tissue model, SAEC, NHLF and HMVEC-L cells were used. All cells were cultured at 37 °C and 5% CO_2_ in primary cell culturing media. After the cells reached 80% confluence, all cell types were subcultured and mixed [30% SAEC, 30% HMVEC-L and 40% NHLF] then dispensed onto a low-attachment 96-well U-bottom plate (Corning, New York, USA). Cells were centrifuged at 600 g for 10min and maintained at 37 °C and 5% CO_2_ in mixed SAGM:EGM-2:FGM-2 media during the experiments. In the various experiments, aggregates were ﻿treated with 1 μg/ml recombinant human Wnt5a and Wnt11 (R&D Systems, Minneapolis, USA) for 72 h.

### Recombinant Lenti (L) viral constructs and hF11 transfection of VEGF-A-GFP construct

VEGF-A sequence was amplified by PCR reaction using forward (5′)’- GGA TTC CTG ACG GAC AGA CAG ACA GAC-3′ and reverse (3′)’- GTC GAC TCA CCG CCT CGG CTT GTC ACA-3′ primer sequences and cloned into Lenti pWPTS vectors. Lentiviral vectors were prepared by co-transfection of three plasmid constructs (envelope construct pMD.G, packaging construct R8.91 and transfer construct pWPTS) into 293 T cells using the calcium-phosphate method as described previously [45]. The HIV-1 derived lentiviral system was kindly provided by Prof. Didier Trono (CMU, Geneva, Switzerland).

Cells were maintained in FGM-2 medium and exposed to lentivirus containing media for 1 h, and then cells were washed and incubated in FGM-2 culturing media. VEGF-A overexpressing F11 cells were then harvested and spheroids were produced as described above. SAEC-F11 VEGF^high^- HMVEC-L and SAEC-F11-HMVEC-L aggregates were cultured for an additional 72 h before RNA isolation or immunfluorescent analysis. Aggregates were exposed to recombinant human Wnt5a and recombinant human Wnt11 (Chinese Hamster Ovary Cell Line, CHO-derived Gln38-Lys380) (R&D Systems, Minneapolis, USA) for 72 h.

### Flow cytometry

3D SAEC-F11 VEGF^high^- HMVEC-L and SAEC-F11-HMVEC-L lung aggregates were cultured for 72 h in the presence or absence of rhWnt5a. Aggregates were then dissociated with Accumax^TM^ (Sigma-Aldrich, St. Louis, USA) solution and washed in PBS once. Single cell suspensions were incubated with Allophycocyanin (APC) conjugated anti-human CD105 (Clone 43A3, BioLegend, San Diego, USA) and Brilliant Violet 421 conjugated anti-human CD31 (Clone VM59, BioLegend, San Diego, USA) for 30min at room temperature in dark. Native lung AC and SCC samples were dissociated by enzymatic digestion (Accumax Solution, Sigma Aldrich, St. Louis, USA) and the single cell suspensions were washed in PBS once, then cells were incubated with APC Cy7 conjugated anti-human CD31 (Clone VM59, BioLegend, San Diego, USA) and APC conjugated CD105 (Clone 43A3, BioLegend, San Diego, USA) antibodies. Cells then were washed in PBS, fixed with 1% PFA and stored at 4 °C in dark until FACS analysis. Labeled cells were analyzed using FACS Canto II flow cytometer (BD Immunocytometry Systems, Erembodegen, Belgium) with BD FACS DIVA software V6 and data were analyzed by FCS Express V3 software.

### 3D sprouting

3D SAEC-F11 VEGF^high^- HMVEC-L and SAEC-F11-HMVEC-L lung aggregates were embedded in 1.5 mg/ml collagen type I solution (BD Biosciences, San Jose, USA) and were cultured for 72 h. Images were taken using Zeiss LSM710 confocal microscope. To identify the migrating cell type; epithelial and endothelial cells were previously cultured with the vital dyes DiD and DiI (Thermo Fisher Scientific, Waltham, USA), respectively, while fibroblast cells remained unstained. Sprouting area containing endothelial cells was determined by Fiji [46], based on the sprout outgrowth.

### PPRE reporter assay

A549 cells were transfected with PPRE-luciferase reporter and PPRE control-luciferase reporter vectors using Lipofectamine 3000 (Thermo Fisher Scientific, Waltham, USA). 6*10^3^ cells were transfected with 100 ng of plasmid DNA mixed with 0.3 μl Lipofectamine 3000 and 0.2 μl P3000 reagent (Thermo Fisher Scientific, Waltham, USA). After overnight incubation, cells were treated with LiCl at 10 mM concentration for 24 h. Luciferase reporter, containing PPRE responsive element can be activated by PPAR activation, which catalyzes luciferin oxyluciferin transformation into luminescent signal. PPAR activation was measured using BrightGlo luciferase assay (Promega, Madison, USA) and detected by Synergy HT plate reader (BioTek, Winooski, USA). Changes in PPAR activation were compared to PPRE control plasmid. RNA isolation was performed after 24 h treatment. Gene expression was compared to non-treated cell cultures. Immunfluorescent staining was performed using purified anti-human VEGF-A antibody (1:100, Clone 26503, R&D Systems, Minneapolis, USA) as primary antibody, visualized by Alexa Fluor 488 conjugated anti-mouse secondary IgG antibody (1:200, Thermo Fisher Scientific, Waltham, USA). Nuclei were stained by TO-PRO3 (Thermo Fisher Scientific, Waltham, USA) and pseudo-colored for blue. Pictures were taken by Zeiss LSM 710.

### PPARgamma agonist and antagonist treatment

F11 cell line was cultured in 24-well plate and was treated with 10 μM rosiglitazone (RSG) and 10 μM GW9662 (Sigma-Aldrich, St. Louis, USA) for 48 h in the presence or absence of rhWnt5a (R&D Systems, Minneapolis, USA). VEGF-A mRNA level was determined using real-time quantitative PCR, while miR-27b expression was measured by Taqman MicroRNA Assay (Thermo Fisher Scientific, Waltham, USA).

### HMVEC-L transwell migration assay

HMVEC-L cells were seeded onto the Transwell insert (8 μm pore size, 6.5 mm diameter) (Corning Costar, Sigma Aldrich, St. Louis, USA) at the density of 2*10^4^. To assess the effect of VEGF-A and Wnt5a, HMVEC-L were cultured in the presence or absence of elevated level of VEGF-A and recombinant human Wnt5a. To gain VEGF-A excess, F11-VEGF^high^ cells were seeded into the well. F11-VEGF^normal^ cells were used as controls. After 24 h, the inserts were stained with Hematoxylin-eosin (detailed protocol below) and pictures were taken using Nikon Eclipse Ti-U inverted microscope (Tokyo, Japan).

### RNA isolation

Total RNA from cell cultures was extracted with MN NucleoSpin RNA isolation kit according to the manufacturer’s protocol (Macherey-Nagel, Düren, Germany). The concentration of RNA samples was measured using NanoDrop (Thermo Fisher Scientific, Waltham, USA).

Total RNA from human lung tissues were obtained using TRIzol reagent (Invitrogen, Thermo Fisher Scientific, Waltham, USA). 1 μg RNA were digested with DNase (Sigma-Aldrich, St. Louis, USA).

### Real-time quantitative PCR

cDNA was synthesized with high capacity RNA to cDNA kit (Thermo Fisher Scientific, Waltham, USA) using 1 μg of total RNA according to manufacturer’s recommendation. Reverse transcription was performed with random hexamer primers. For gene expression analysis, quantitative RT-PCR was performed using SensiFAST SYBR Green reagent (BioLine, London, UK). Amplifications were run on ABI StepOnePlus system. Gene expressions were analyzed with StepOne software and normalized to beta-actin housekeeping gene. The primer sequences are shown in Table 1. PCR conditions were set as follows: one cycle 95 °C for 2min, 40 cycles at 95 °C for 5 s and 60 °C for 30 s. Changes in gene expression were calculated according to the 2^-ddCt^ method.

**Table 1:** Quantitative real-time PCR primers

Primers	Forward	Reverse
beta-actin	GCGCGGCTACAGCTTCA	CTTAATGTCACGCACGATTTCC
PPARgamma	GCTTTTGGCATACTCTGTGATCTC	GGTGGCCATCCGCATCT
VEGF-A	GGGCAGAATCATCACGAAGT	TGGTGATGTTGGACTCCTCA
CD105	CTCTCCAGGCATCCAAGCAA	CAGGCTGGAATTGTAGGCCA
CD31	GCTGACCCTTCTGCTCTGTT	ATCTGGTGCTGAGGCTTGAC
HIF-1alpha	GCCAGACGATCATGCAGCTA	ATCCATTGATTGCCCCAGC
IL-1beta	TCAGCCAATCTTCATTGCTCAA	TGGCGAGCTCAGGTACTTCTG
Wnt5a	CAAAGCAACTCCTGGGCTTA	CCTGCTCCTGACCGTCC

### TaqMan microRNA assay

Reverse transcription reaction was set up with ABI TaqMan microRNA assay kit (Thermo Fisher Scientific, Waltham, USA) using 100 ng of total RNA according to manufacturer’s recommendations. Each reaction contains specific miR-27b, miR-200b and U6 primers. PCR reaction was performed using TaqMan MicroRNA Assay (20x), TaqMan Universal Master Mix (2x) (Thermo Fisher Scientific, Waltham, USA) and product from reverse transcriptase reaction. TaqMan PCR reaction was performed using ABI StepOnePlus system and data were analyzed with StepOne software. PCR conditions set as follows: one cycle 95 °C for 10min, 40 cycles at 95 °C for 15 s and 60 °C for 60 s. MicroRNA expression was normalized to U6 expression.

### Sections

Mice were anaesthetized with sodium pentobarbital intraperitoneally and lungs were filled up with 1:1 ratio of PBS:cryostate embedding media (TissueTek, Alphen aan den Rijn, Netherland), and frozen down at −80 °C. Human samples were collected in PBS containing 1% of FBS and then were filled up with PBS:cryostate embedding media and kept at −80 °C until processing. The 3D lung aggregates were carefully removed from the 96-well plates and embedded into TissueTek embedding media and immediately frozen down at −80 °C. For histological staining 8 μm thick cryostat sections were cut and fixed in 4% PFA for 20min.

### Hematoxylin eosin staining

Eight micrometers thick cryostat sections or Transwell inserts (Corning, New York, USA) were cut and stained in Mayer’s hematoxylin solution (Sigma-Aldrich, St. Louis, USA) for 10min. Sections were washed in running tap water for 10min, then differentiated with 0.25% acetic acid (Sigma Aldrich, St. Louis, USA) for 1min. After the differentiation step, slides were washed with tap water and stained in eosin solution for 2min, then washed. Sections were mounted using Vectashield mounting medium (Vector Laboratories, Burlingame, USA). Images were taken using Nikon Eclipse Ti-U inverted microscope (Tokyo, Japan).

### Antibodies, fluorescent and immunohistochemical staining

Paraffin embedded lung AC and SCC samples were stained with a routine IHC staining procedure using Vision Biosystems bondTM automated immunostainer (Leica, Wetzlar, Germany). Primary antibody of rat monoclonal anti-human Wnt5a (Clone 442625, R&D Systems, Minneapolis, USA) was used in 1:100 dilution. Cryostat sections were fixed. Fixed slides were rehydrated and blocked for 20min in 5% BSA (Sigma Aldrich, St. Louis, USA) in PBS. For mouse sections, Alexa Fluor 594 conjugated monoclonal anti-mouse CD31 (1:100, Clone MEC13.3, BioLegend, San Diego, USA), Alexa Fluor 488 conjugated monoclonal anti-mouse CD105 (1:100, Clone MJ7/18, BioLegend, San Diego, USA), purified monoclonal anti-mouse VEGF-A (1:100, Clone 1 F07-2C01, BioLegend, San Diego, USA) and purified monoclonal anti-mouse Wnt5a (1:50, Clone 442625, R&D Systems, Minneapolis, USA) primary antibodies were applied. For 3D lung tissue samples, the sections were stained with purified monoclonal anti-human CD31 antibody (1:100, Clone WM59, BioLegend, San Diego, USA). To detect VEGF-A expression in A549 and F11 cell line, purified monoclonal anti-human VEGF-A antibody (1:100, Clone 26503, R&D Systems, Minneapolis, USA) was used. The secondary antibodies were Alexa Fluor 488 or 555 conjugated anti-mouse IgG antibodies (1:200, Thermo Fisher Scientific, Waltham, USA), respectively. The nuclei were counterstained with TO-PRO-3 (1:1000, Thermo Fisher Scientific, Waltham, USA) and showed in blue as pseudo-color blue. Pictures were captured using Zeiss LSM 710 microscope (Zeiss, Oberkochen, Germany) equipped with analysis software. Images and fluorescent intensity were measured with Fiji software [46]. Intensity of two groups was analyzed with the independent samples *t*-test.

### Statistical analysis

Statistical analysis was performed with SPSS version 20 software. Data are presented as mean ± standard error of mean (SEM), and statistical analysis was performed using the independent samples *t*-test and one-way ANOVA with Bonferroni correction. *p* < 0.05 was considered as significant.

## Results

### PPARgamma regulates VEGF-A expression

**Fig. 1:**
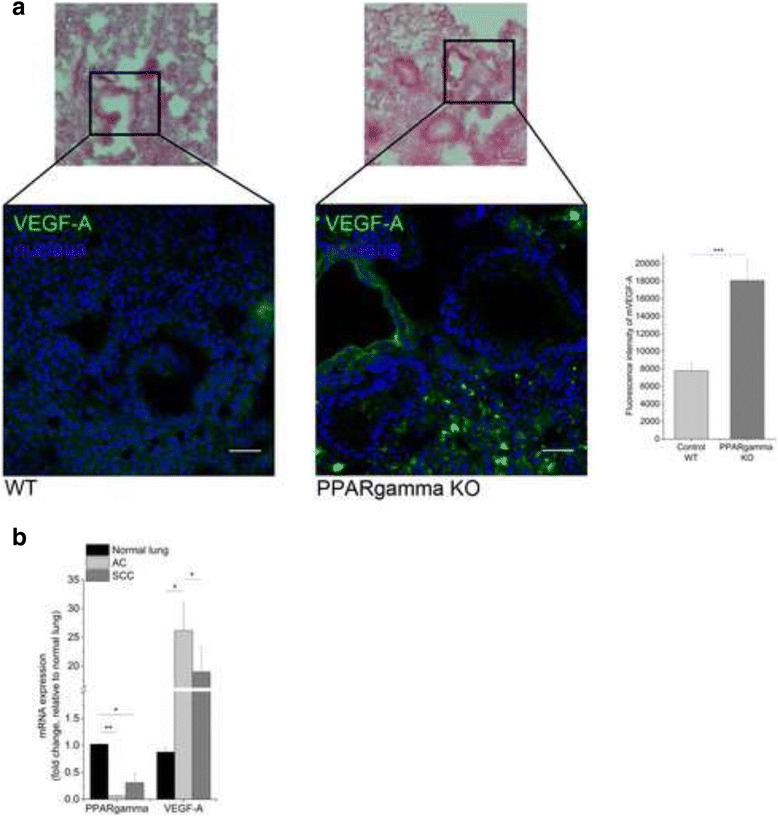
VEGF-A expression in PPARgamma KO mice and human lung tumors. **a,** Hematoxylin eosin staining and immunofluorescent staining of the lung of wild type C57BL/6 and PPARgamma knock-out (KO) mice show a significantly increased level of mVEGF-A in KO lung tissues. Scale bars, 200 μm and 50 μm. Intensity data are representation of three independent experiments as mean ± SEM. **b,** Resected human lung AC and SCC revealed significant PPARgamma decrease and VEGF-A expression increase. Data are presented as mean ± SEM. One-way ANOVA, post hoc Bonferroni; *n* = 11 and *n* = 12 per groups. *P* < 0.05 was considered as significant, * *p* < 0.05, ** *p* < 0.01, *** *p* < 0.001

To clarify the role of PPARgamma in regulation of VEGF-A expression, lungs of PPARgamma knock-out mice were studied. Fluorescent immunohistochemistry of VEGF-A protein revealed a significantly higher expression of the VEGF-A protein in the lungs of PPARgamma KO mice than in their wild-type litter mates (Fig. 1a), indicating that PPARgamma inactivation is required for VEGF-A production. Emphasizing the initial observation, significantly (*p* < 0.05) increased VEGF-A expression was detected in primary clinical samples (Additional file 2: Table S1) of both AC and SCC (Fig. 1b), while PPARgamma levels were reduced (*p* < 0.05 and *p* < 0.01, respectively) in both tumor types compared to normal, non-diseased, primary lung controls. Additionally, comparative analysis of primary AC and SCC samples highlighted existing differences in the two NSCLC subtypes. While lower PPARgamma mRNA levels characterized significantly higher VEGF-A expression in AC, higher PPARgamma and lower VEGF-A mRNA described SCC (Fig. 1b).

**Fig. 2:**
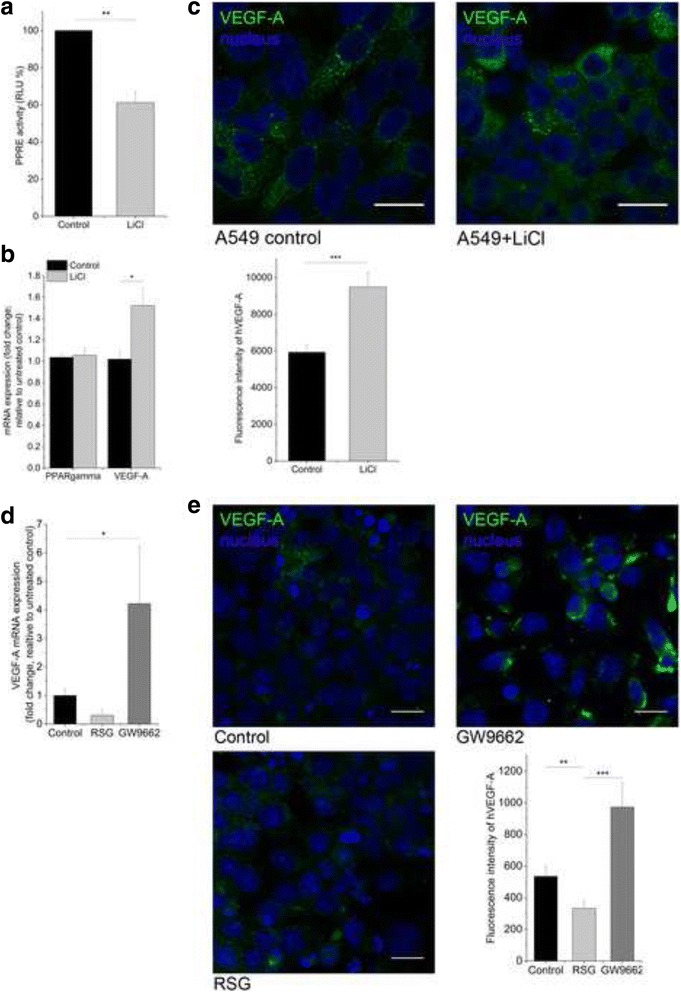
VEGF-A expression following modification of PPARgamma activity in A549 lung adenocarcinoma cell line tranfected with PPRE control or reporter plasmid. **a,** Mimicking beta-catenin dependent canonical Wnt pathway activation using 10 mM LiCl led to significant decrease in PPRE reporter activity. **b,** 10 mM LiCl treatment induced VEGF-A mRNA expression compared to PPRE control cells and also **c,** 10 mM LiCl increased VEGF-A protein levels. Error bars, SEM. One-way ANOVA, post hoc Bonferroni; *n* = 4. Scale bars, 20 μm. **d,** VEGF-A mRNA expression decreased after 10 μM PPARgamma agonist treatment (RSG), while 10 μm PPARgamma specific antagonist (GW9662) increased VEGF-A transcript levels. Independent samples *t*-test, *n* = 3. **e,** VEGF-A protein level shows similar pattern after 10 μm RSG and 10 μm GW9662 treatment. Fluorescence intensity are representations of three different experiments as mean ± SEM. One-way ANOVA, post hoc Bonferroni; *n* = 3. Scale bars, 20 μm. *P* < 0.05 was considered as significant, * *p* < 0.05, ** *p* < 0.01, *** *p* < 0.001

To prove that canonical Wnt signaling induced downregulation of PPARgamma triggers VEGF-A expression, A549 lung adenocarcinoma cell line was treated with the chemical activator of the canonical Wnt signaling pathway, the beta-catenin activator LiCl [41]. Inhibition of PPAR reporter activity (Fig. 2a) by LiCl (10 mM) lead to increased VEGF-A mRNA (Fig. 2b) and protein expression (Fig. 2c). Inhibition of PPARgamma by LiCl did not affect PPARgamma mRNA expression suggesting that PPARgamma activity is the key factor in VEGF-A regulation.

To support this observation and as the reporter system is not specific to PPARgamma, we used direct PPARgamma agonist and antagonist treatment of human fibroblast cells to determine the role of PPARgamma in VEGF-A regulation. Inhibition of PPARgamma upon GW9662 antagonist treatment increased VEGF-A levels, while the presence of a PPARgamma agonist resulted in decreased VEGF-A mRNA and protein levels (Fig. 2d and e) supporting the theory that VEGF-A upregulation in AC and SCC lung cancers are a direct consequence of PPARgamma reduction.

### Wnt5a induces miR-27b a regulator of both PPARgamma expression and blood vessel branching

**Fig. 3:**
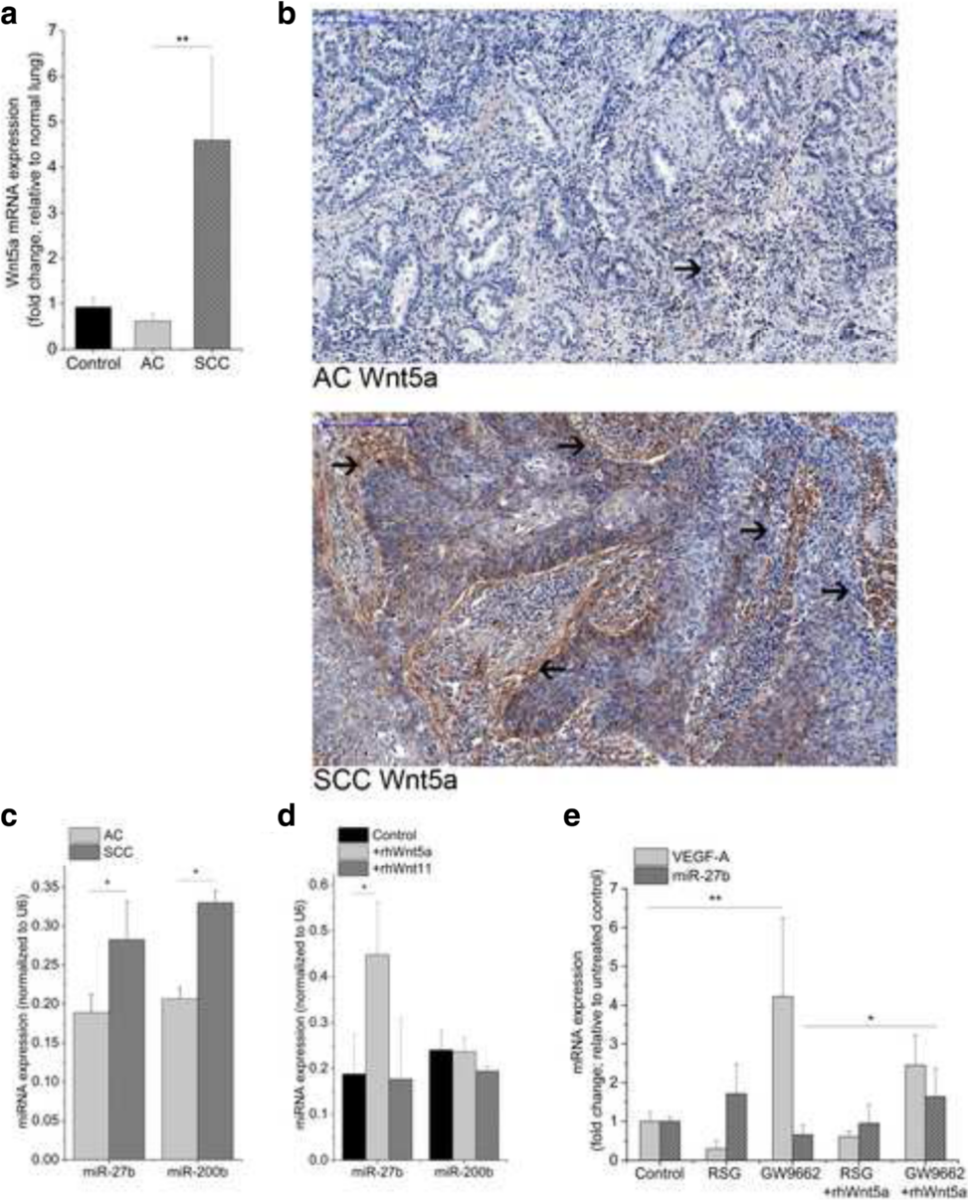
Transcript analysis of Wnt5a, miRNA and VEGF-A in primary human lung cancer samples of AC and SCC and 3D in vitro lung aggregate cultures. **a,** Wnt5a mRNA is significantly upregulated in SCC compared to both normal lung and AC specimens. Error bars, SEM. One-way ANOVA, post hoc Bonferroni; *n* = 11 and *n* = 12 per groups. **b,** Immunohistochemical staining of Wnt5a in primary resected AC and SCC samples, *n* = 3 per groups. **c,** miR-27b and miR-200b expression levels are significantly lower in AC compared to SCC. Error bars; SEM. Independent samples *t*-test, *n* = 5 per groups. **d,** miR-27b is up-regulated by rhWnt5a in 3D lung aggregate cultures, while neither miR-27b nor miR-200b was affected by rhWnt11. Error bars; SEM. One-way ANOVA, post hoc Bonferroni, *n* = 6. **e,** VEGF-A and miR-27b expression levels after 10 μM RSG (PPARgamma agonist) and 10 μM GW9662 (PPARgamma antagonist) and combination treatment with rhWnt5a. Error bars; SEM. Independent samples *t*-test *n* = 3. *P* < 0.05 was considered as significant, * *p* < 0.05, ** *p* < 0.01, *** *p* < 0.001

As only the beta-catenin dependent canonical Wnt signaling has been reported repeatedly to down-regulate PPARs [30, 44] and consequently up-regulate VEGF-A [47], it was not clear how SCC samples with high levels of non-canonical Wnt ligands (Fig. 3a and b) can trigger the same mechanism? In the literature PPARgamma has been described to have a highly conserved binding site in its 3′UTR that is a direct target of miR-27b [48]. Binding of miR-27b to its target sequence can suppress PPARgamma expression [49] and augment VEGF-A induced angiogenesis [50]. Importantly, both miR-27b as well as another miRNA, miR-200b can inhibit blood vessel branching during angiogenesis [51, 52]. Based on the above studies we theorized that differences in formation of blood vessel networks in AC and SCC are due to variations in microRNAs and VEGF-A levels. To investigate, both miR-27b and miR-200b levels were measured in primary human AC and SCC samples. Significantly higher expression levels were determined for both miRNA in primary SCC tissues compared to AC (Fig. 3c) indicating existence of miRNA dependent differential regulation of angiogenesis in the two NSCLC subtypes. To test whether the different molecular microenvironment characterized by increased Wnt5a levels in SCC would explain variations in miRNA expression, 3D lung aggregate cultures were exposed to non-canonical Wnt-s; rhWnt5a and rhWnt11. Wnt5a and Wnt11 were selected in the experiments as they were both reported to have higher levels in SCC than in AC [37]. Interestingly, while rhWnt11 had no effect on either miRNAs (Fig. 3d), miR-27b expression was significantly increased by rhWnt5a treatment (Fig. 3d), while miR-200b levels were unaffected. Remarkably, rhWnt5a could not up-regulate miR-27b in the presence of a PPARgamma agonist, only if the antagonist was present (Fig. 3e). The actual VEGF-A expression was, however, lower (Fig. 3e) than induced by the PPARgamma antagonist on its own. Additionally, while rhWnt5a treatment on its own did not increase VEGF-A levels at the time point analyzed (48 h treatment, data not shown) implicating a parallel mechanism that is required for PPARgamma inhibition even in the presence of the non-canonical Wnt pathway dominated microenvironment.

### Wnt5 regulates VEGF-A induced endothelial cell motility

**Fig. 4:**
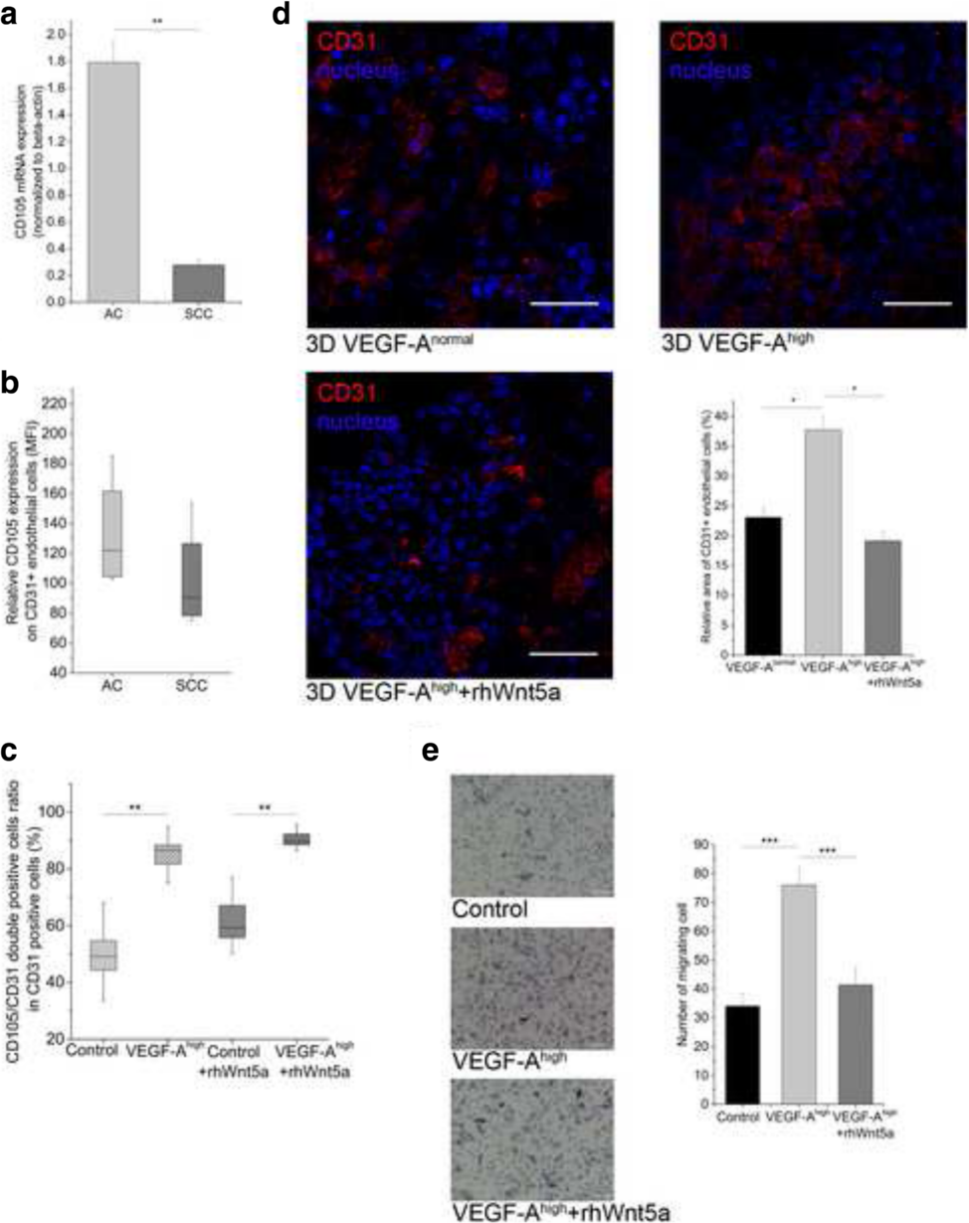
The effect of Wnt5a on VEGF-A induced endothelial cell activation and motility. **a,** CD105 mRNA expression is significantly higher in primary AC compared to SCC samples. Error bars, SEM. One-way ANOVA, post hoc Bonferroni; *n* = 11 and *n* = 12 per groups. **b,** Flow cytometric analysis of CD105 protein expression in CD31 positive endothelial cells in primary AC and SCC samples. *n* = 6 per groups. **c,** Flow cytometric analysis of CD105 levels in normal and high VEGF-A microenvironment in 3D lung aggregate tissues has also shown an increase of activation marker CD105 in VEGF-A^high^ tissues. The double positive (CD105/CD31) cell population was considered as activated endothelial cells. Independent samples *t*-test, *n* = 6. 1 μg/ml rhWnt5a treatment had no effect on the VEGF-A induced endothelial cell activation measured by the double positive (CD105/CD31) cell population identified by flow cytometric analysis. Independent samples *t*-test, *n* = 6. **d,** Localization of endothelial cells was identified by immunoflurescent staining of CD31 and analyzed by confocal microscopy in 3D lung tissue aggregates. In VEGF-A^normal^ microenvironment endothelial cells remained diffuse in the tissue. Under VEGF-A excess endothelial cell migrated towards the source (VEGF-A^high^ fibroblasts) of the signal in the center of the aggregate tissue. 1 μg/ml rhWnt5a treatment of VEGF-A^high^ tissue aggregates inhibited endothelial cell accumulation in the center of the aggregate. Bar chart represents the quantification of endothelial cell distribution. Relative area of CD31+ endothelial cells are compared to total field. Percentages were calculated as relative area of endothelial cells/area of total field *100. Error bars; SEM. Independent samples *t*-test *n* = 3. Representative images of three independent experiments are shown. Scale bars, 50 μm. **e**, HMVEC-L transwell migration assay. Endothelial cells migrate significantly faster towards VEGF-A^high^ fibroblast, while 1 μg/ml rhWnt5a can reverse the effect of elevated VEGF-A level. Scale bar 100 μm. One-way ANOVA, *n* = 3. *P* < 0.05 was considered as significant, * *p* < 0.05, ** *p* < 0.01, *** *p* < 0.001

As VEGF-A has previously been determined to stimulate endothelial cell proliferation and migration [53], expression of endoglin (Eng or CD105) a transmembrane auxillary receptor for transforming growth factor-beta (TGF-beta) that is predominantly expressed on proliferating endothelial cells was also tested [54]. Analysis of primary SCC and AC tissue samples revealed that the endothelial cell proliferation marker Eng (CD105) was significantly lower in SCC compared to AC (Fig. 4a and b). Although VEGF-A was significantly higher than normal in both cancer subtypes, VEGF-A levels in SCC were lower than in AC (Fig. 1b). To be able to study the molecular effects of high VEGF-A levels on endothelial cells, in vitro studies were performed using a three dimensional (3D) human lung aggregate model tissue consisting of primary human small airway epithelial cells (SAEC), microvascular lung endothelial cells (HMVEC-L), and human fibroblasts (NHLF and F11) (Additional file 2: Figure S1). The 3D aggregate culture conditions provided close to natural, yet defined, cellular environment for molecular studies. qRT-PCR analysis of the lung model tissue aggregates revealed highly similar expression levels of angiogenic stimulators including VEGF-A, IL-1beta and HIF-1alpha to primary human lungs (Additional file 3: Figure S2) indicating that the model is suitable to study pro- and/or anti-angiogenic mechanisms. To recreate the VEGF-A high tumor-like microenvironment VEGF-A^165^ was cloned into human fibroblast cells (Additional file 4: Figure S3). The effects of VEGF-A were investigated by comparing aggregates with VEGF-A^normal^ and VEGF-A over-expressing (VEGF-A^high^) fibroblasts (Additional file 5: Figure S4). Flow cytometric analysis revealed that high levels of VEGF-A lead to elevated Eng (CD105) expression on CD31^+^ endothelial cells (Fig. 4c, Additional file 6: Figure S5). To test whether Wnt5a can modulate VEGF-A^high^ microenvironment, 3D lung tissue aggregates containing VEGF-A^normal^ and VEGF-A^high^ fibroblasts were exposed to rhWnt5a. Flow cytometric analysis revealed that rhWnt5a did not block proliferation marker Eng (CD105) expression (Fig. 4c, Additional file 6: Figure S5) indicating that Eng (CD105) is not under Wnt5a control.

This result was surprising. Because the endothelial cell proliferation marker Eng (CD105) expression was lower in SCC compared to AC, and VEGF-A levels were similarly elevated in both cancer types, endothelial cell proliferation would be expected. To investigate, lung tissues of control and PPARgamma KO mice were stained for CD105. Significantly lower levels of CD105 was detected in PPARgamma KO animals than in their wild type controls (Additional file 7: Figure S6) indicating that PPARgamma associated events are responsible for the reduction but not Wnt5a. As Wnt5a levels are not affected in lungs of the PPARgamma KO animals (Additional file 8: Figure S7), our findings further suggest that CD105 expression is not under Wnt5a control. To clarify the role of Wnt5a, cellular motility was investigated in the model cultures. All these findings led us to the hypothesis that endothelial activity cannot be the only answer to the different therapeutic response. As SCC possesses more vulnerable capillaries [16] and lower microvessel density [15], it might be an indication of an altered endothelial behavior, especially in migration and motility.

In the VEGF-A^high^ microenvironment endothelial cells migrated towards the source of VEGF-A, the VEGF-A^high^ fibroblasts (Fig. 4d and Additional file 5: Figure S4). As fibroblasts naturally provide the core of the 3D lung tissue aggregate co-cultures, endothelial cells concentrated in the center of the tissue aggregate. In the aggregate tissues with normal VEGF-A levels the endothelial cells remained evenly distributed (Fig. 4d). To investigate whether Wnt5a affects endothelial cell motility, aggregate cultures were treated with rhWnt5a. This inhibited endothelial cell migration towards VEGF-A^high^ fibroblasts, and endothelial cells remained scattered amongst other cell types in the aggregate lung tissue. The inhibitory effect of Wnt5a on endothelial cell motility was tested using migration assays in transwell chambers. HMVEC-L cells migrated significantly faster towards VEGF-A^high^ fibroblasts, while addition of recombinant human Wnt5a significantly inhibited VEGF-A induced endothelial cell migration (Fig. 4e).

## Discussion

While cancer progression is frequently attributed to increased angiogenesis, molecular regulation that can lead to significant differences in anti-angiogenic therapy responses have not been reported. As AC and SCC have characteristic molecular differences in their Wnt microenvironment [37] and differential treatment responses to anti-angiogenic therapy [13] including increased necrosis and pulmonary hemorrhage in SCC, comparative studies of the two NSCLC subtypes provided a promising platform for molecular studies of blood vessel formation. Recent evidence suggested that adverse reactions in SCC patients might be due to malfunction of endothelial repair processes [55], tumor type specific erosion of blood vessels [56] or involvement of major vascular structures in SCC [57]. Our studies provided an additional dimension to the potential mechanism by making extensive use of primary human lung tissues both of normal lung and resected lung AC and SCC tumors, knock-out mice as well as an in vitro 3D human lung tissues model assembled to assess the role of Wnt5a in angiogenesis. Based on our study, we postulate that decreased expression of PPARgamma is primarily responsible for induction of increased VEGF-A levels detected both in AC and SCC. However, the molecular mechanism that leads to PPARgamma down-regulation is different in AC and SCC, and it is this molecular mechanism rather than tumor localization that leads to more serious adverse reactions in SCC patients.

This conclusion is based on several pieces of evidence presented here. Down-regulation of PPARgamma was demonstrated to be essential for up-regulation of the angiogenesis stimulator factor VEGF-A using PPARgamma KO animals, cell lines and a fully human in vitro lung tissue aggregate system where the PPRE reporter system, application of PPARgamma agonist and antagonist supported direct regulatory interactions between the two molecules. Consequently, the significantly reduced PPARgamma and increased VEGF-A levels in both primary AC and SCC explained increased vascularization in both tumors. Differential therapeutic response, however, could not be explained by the very similar molecular microenvironments of the two tumor subtypes.

**Fig. 5:**
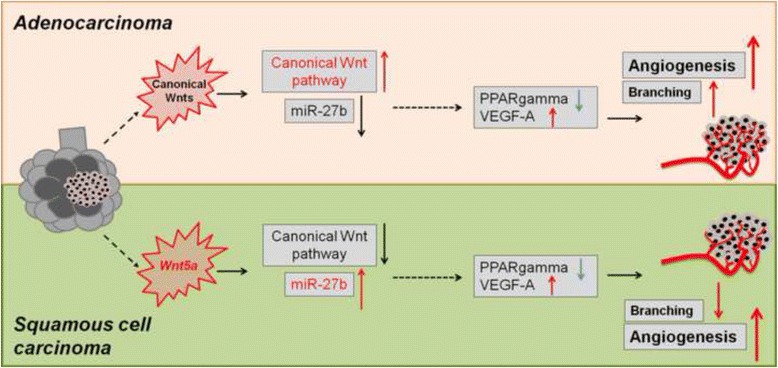
Summary of the role of Wnt dependent regulation of PPARgamma in tumor angiogenesis

For better understanding of the process of PPARgamma down-regulation we considered that down-regulation of PPARgamma transcription is a beta-catenin-dependent and therefore canonical Wnt pathway dependent process [44]. As lung ACs are characterized by increased canonical and therefore beta-catenin dependent Wnt signal activity [58] such molecular basis can explain PPARgamma reduction in AC [44]. The similarly high VEGF-A and reduced PPARgamma levels in SCC samples seem counter-intuitive as in SCC the non-canonical, beta-catenin independent Wnts play the dominant role [59]. Our initial data, however, provide some explanations. Wnt5a is the characteristically highly expressed Wnt in cancers with squamous histology and increased Wnt5a levels are also a trademark of lung SCC. Interestingly, Wnt5a can up-regulate miR-27b which miRNA has been reported to bind to the highly conserved binding site in the 3′UTR of PPARgamma [48] and suppress its expression [49]. Consequently, up-regulation of VEGF-A [50] can use this particular route (Fig. 5). Wnt5a did not induce VEGF-A directly at the time-points measured in this study, indicating that other mechanisms are also needed to achieve inhibition of PPARgamma. Inhibition of PPARgamma in the presence of Wnt5a, however, modified VEGF-A levels supporting our theory that Wnt5a plays a modulatory role in the angiogenic process. Certainly, there are limitations to the study as not all VEGF-A isoforms [60] nor pericytes were investigated in the above experiments therefore the full effect of Wnt5a on the angiogenic process could not be assessed. However, since Cox-2 (an enzyme responsible for pericyte recruitment) is inhibited by PPARgamma [61, 62], we can hypothesize that lower PPARgamma levels and/or activity could reduce pericyte coverage. As SCC has higher PPARgamma levels than AC, perhaps high Wnt5a can not only reduce endothelial cell but also pericyte mobility resulting in immature vessel formation that makes SCC more prone to hemorrhage. Further studies are needed to investigate the above hypothesis.

Despite the limitations, further scrutiny of the data highlighted additional differences between AC and SCC at the level of blood vessel branching. Inhibitors of branching and tube formation like miR-200b [63] and miR-27b [51] are both increased in SCC compared to AC potentiating differences in the actual blood vessel formation and pattern in tumors of squamous histology. Such differences could lead to alterations in therapeutic response. As only miR-27b is regulated by Wnt5a, further studies are needed to find the molecules that control miR-200b. Additionally, miR-27b can also block the Wnt5a target NF-kappaB [64] and the TGF-beta target, Gremlin-1 [65]. As VEGF-A is also upregulated following by NF-kappaB activation [66] and Gremlin-1 acts as a pro-angiogenic factor via binding to VEGFR2 [67] the lower VEGF-A levels in SCC might explain such differences and demonstrate a very complex regulatory process of angiogenesis that requires follow-up investigations to provide explanations to more severe adverse therapeutic reactions in SCC.

## Conclusion

Finally, it may be possible in the future to use serum levels of Wnt5a, miR-27b, PPARgamma levels and/or activity of the tumor tissue as prognostic markers to identify groups of patients that are at higher risk of developing hemorrhage if treated with anti-angiogenic therapies.

## Additional files

Additional file 1:
**Table S1.** Characteristics of patients. The table contains number, histological types, sex, age and pathological TNM status of all patients. (DOCX 14 kb)
Additional file 2:
**Figure S1.** 3D human lung tissue aggregates to study angiogenesis. To investigate the molecular background of angiogenesis, a three dimensional human lung tissue model was set up using three characteristic cell types of the lung. Namely, primary normal small airway epithelial cells (SAEC), normal human lung fibroblast (NHLF) or VEGF-A-GFP overexpressing human fibroblast (F11) and human microvascular endothelial cells lung subtype (HMVEC-L). GFP aided the visualization of VEGF-A overexpressing fibroblasts in the core of the lung model. Actin was labeled by Alexa Fluor 568 conjugated phalloidin (Thermo Fisher Scientific, Waltham, USA) to detect all cells in the tissue. Scale bar, 200 μm. (TIF 1897 kb)
Additional file 3:
**Figure S2.** Analysis of expression of angiogenic genes in 3D lung aggregates and normal human lung. Comparing the in vitro model to normal human lung tissue revealed that co-culture expresses VEGF-A, IL-1beta and HIF-1alpha angiogenic factors at the same level. qRT-PCR data are normalized to beta-actin housekeeping gene. Error bars, SEM. Independent samples *t*-test, *n* = 3. *P* < 0.05 was considered as significant, ns: not significant. (TIF 90 kb)
Additional file 4:
**Figure S3.** Overexpression of VEGF-A in F11 cell line. Human VEGF-A-IRES-GFP was cloned into human F11 cell line. Overexpression of hVEGF-A protein was analyzed by fluorescent staining using anti-human VEGF-A as primary antibody. The secondary antibody was Northern Light anti-mouse NL-557. As VEGF-A is a secreted, soluble factor, cells were treated with brefeldin-A at 10 μg/ml for 4 h to inhibit protein transport. Accumulation of the protein was detected after brefeldin-A treatment. Images were captured using Olympus IXB1 fluorescence microscopy equipped with CCD camera. Scale bars, 50 μm. (TIF 3030 kb)
Additional file 5
**Figure S4.** Area of sprout outgrowth from 3D lung aggregates in normal and high VEGF-A microenvironment. Endothelial cells are evenly distributed in VEGF-A^normal^ microenvironment, while in VEGF-A^high^ microenvironment the endothelial cells remained close to the VEGF-A source. Data are representation of three independent experiments. Independent samples *t*-test. Scale bars, 200 μm. (TIF 1597 kb)
Additional file 6:
**Figure S5.** Flow cytometric analysis of VEGF-A overexpressing 3D lung aggregates in the absence or presence of rhWnt5a. Representative dot plots of flow cytometric analysis of five independent experiments. 3D tissue cultures were created using normal VEGF-A and VEGF-A^high^ fibroblasts, respectively. Then the cultures were incubated in the presence or absence of rhWnt5a, dissociated and stained with anti-CD105 and anti-CD31 antibodies and analyzed in a flow cytometer. (TIF 469 kb)
Additional file 7:
**Figure S6.** Endoglin (CD105) expression of CD31+ endothelial cells in the lung of WT and PPARgamma KO mice. Immunfluorescence staining of wild type and PPARgamma KO mice showed decreased level of CD105 protein expression in PPARgamma KO lung tissues. Intensity data are representation of three individual experiments. Scale bars 50 μm. Staining intensity was quantified and presented in a bar chart. Error bars, SEM. Independent samples *t*-test, *n* = 3. *P* < 0.05 was considered as significant, *P* < 0.001. (TIF 3853 kb)
Additional file 8:
**Figure S7.** Wnt5a expression in the lung of WT and PPARgamma KO mice. No differences were detected in Wnt5a protein expression in wild type and PPARgamma KO mice. (Scale bars 50 μm). The stainings of lung tissue sections are representatives of three individual experiments. Fluorescence intensity was quantified and presented in a bar chart. Error bars, SEM. Independent samples *t*-test, *n* = 3. *P* < 0.05 was considered as significant, ns: not significant. (TIF 769 kb)

## Abbreviations

AC: Adenocarcinoma
APC: Allophycocyanine
bFGF: Basic fibroblast growth factor
EGM-2 MV: Microvascular endothelial cell growth medium
FGM-2: Fibroblast growth medium
HMVEC-L: Human lung microvascular endothelial cell
LC: Lung cancer
NHLF: Normal human lung fibroblast
NSCLC: Non small cell lung cancer
PBS: Phosphate buffered saline
PCP: Planar cell polarity
PDGF: Platelet derived growth factor
PFA: Paraformaldehyde
PPAR: Peroxisome proliferator-activated receptor
PPARgamma KO: PPARgamma knock-out
PPRE: PPAR response element
qRT-PCR: Quantitative real-time polymerase chain reaction
RSG: Rosiglitazone
SAEC: Small airway epithelial cell
SAGM: Small airway cell growth medium
SCC: Squamous cell carcinoma
SCLC: Small cell lung cancer
VEGF: Vascular endothelial growth factor

## References

[1] Jemal A, Siegel R, Ward E, Hao Y, Xu J, Thun MJ (2009). Cancer statistics. CA. Cancer J. Clin. [Internet]..

[2] Ettinger DS, Akerley W, Borghaei H, Chang AC, Cheney RT, Chirieac LR, et al. Non-small cell lung cancer. J Natl Compr Canc Netw. [Internet] 2012;10:1236–71. Available from: https://www.ncbi.nlm.nih.gov/pubmed/23054877. 10.6004/jnccn.2012.0130 23054877

[3] Molina JR, Yang P, Cassivi SD, Schild SE, Adjei AA (2008). Non-small cell lung cancer: epidemiology, risk factors, treatment, and survivorship. Mayo Clin. Proc. [Internet].

[4] D’Addario G, Früh M, Reck M, Baumann P, Klepetko W, Felip E (2010). Metastatic non-small-cell lung cancer: ESMO Clinical Practice Guidelines for diagnosis, treatment and follow-up. Ann. Oncol. [Internet].

[5] Stahel R, Peters S, Baas P, Brambilla E, Cappuzzo F, De Ruysscher D (2013). Strategies for improving outcomes in NSCLC: a look to the future. Lung Cancer [Internet].

[6] Vogelstein B, Papadopoulos N, Velculescu VE, Zhou S, Diaz LA, Kinzler KW (2013). Cancer genome landscapes. Science [Internet].

[7] Boolell V, Alamgeer M, Watkins D, Ganju V (2015). The evolution of therapies in non-small cell lung cancer. Cancers (Basel). [Internet].

[8] Korpanty G, Smyth E, Sullivan LA, Brekken RA, Carney DN (2010). Antiangiogenic therapy in lung cancer: focus on vascular endothelial growth factor pathway. Exp. Biol. Med. (Maywood). [Internet].

[9] Moens S, Goveia J, Stapor PC, Cantelmo AR, Carmeliet P (2014). The multifaceted activity of VEGF in angiogenesis - Implications for therapy responses. Cytokine Growth Factor Rev. [Internet].

[10] Ranieri G, Patruno R, Ruggieri E, Montemurro S, Valerio P, Ribatti D (2006). Vascular endothelial growth factor (VEGF) as a target of bevacizumab in cancer: from the biology to the clinic. Curr. Med. Chem. [Internet].

[11] Zhan P, Wang J, Lv X, Wang Q, Qiu L, Lin X (2009). Prognostic value of vascular endothelial growth factor expression in patients with lung cancer: a systematic review with meta-analysis. J. Thorac. Oncol. [Internet].

[12] Sandler A, Gray R, Perry MC, Brahmer J, Schiller JH, Dowlati A (2006). Paclitaxel-carboplatin alone or with bevacizumab for non-small-cell lung cancer. N. Engl. J. Med. [Internet]..

[13] Piperdi B, Merla A, Perez-Soler R (2014). Targeting angiogenesis in squamous non-small cell lung cancer. Drugs [Internet].

[14] Oliver TG, Patel J, Akerley W (2015). Squamous non-small cell lung cancer as a distinct clinical entity. Am. J. Clin. Oncol. [Internet].

[15] Kojima H, Shijubo N, Abe S (2002). Thymidine phosphorylase and vascular endothelial growth factor in patients with Stage I lung adenocarcinoma. Cancer [Internet].

[16] Yazdani S, Miki Y, Tamaki K, Ono K, Iwabuchi E, Abe K (2013). Proliferation and maturation of intratumoral blood vessels in non-small cell lung cancer. Hum. Pathol. [Internet]..

[17] Pan H, Clarke B, Wickline SA. Macrophage pro-angiogenic miRNA, miR-27b, is under NF-kB transcription regulation. FASEB J. 2012;26:1120.9–1120.9. Federation of American Societies for Experimental Biology.

[18] Pecot CV, Rupaimoole R, Yang D, Akbani R, Ivan C, Lu C (2013). Tumour angiogenesis regulation by the miR-200 family. Nat. Commun. [Internet].

[19] Vasudev NS, Reynolds AR (2014). Anti-angiogenic therapy for cancer: current progress, unresolved questions and future directions. Angiogenesis [Internet].

[20] Xin X, Yang S, Kowalski J, Gerritsen ME (1999). Peroxisome proliferator-activated receptor gamma ligands are potent inhibitors of angiogenesis in vitro and in vivo. J. Biol. Chem. [Internet]..

[21] Jiménez R, Sánchez M, Zarzuelo MJ, Romero M, Quintela AM, López-Sepúlveda R (2010). Endothelium-dependent vasodilator effects of peroxisome proliferator-activated receptor beta agonists via the phosphatidyl-inositol-3 kinase-Akt pathway. J. Pharmacol. Exp. Ther. [Internet].

[22] Peeters LLH, Vigne J-L, Tee MK, Zhao D, Waite LL, Taylor RN (2006). PPAR $\upgamma$ represses VEGF expression in human endometrial cells: Implications for uterine angiogenesis. Angiogenesis [Internet].

[23] Gacche RN (2015). Compensatory angiogenesis and tumor refractoriness. Oncogenesis [Internet].

[24] Margeli A, Kouraklis G, Theocharis S (2003). Peroxisome proliferator activated receptor-gamma (PPAR-gamma) ligands and angiogenesis. Angiogenesis [Internet].

[25] Jerkic M, Rivas-Elena JV, Prieto M, Carrón R, Sanz-Rodríguez F, Pérez-Barriocanal F (2004). Endoglin regulates nitric oxide-dependent vasodilatation. FASEB J. [Internet].

[26] Lebrin F, Goumans M-J, Jonker L, Carvalho RLC, Valdimarsdottir G, Thorikay M (2004). Endoglin promotes endothelial cell proliferation and TGF-beta/ALK1 signal transduction. EMBO J. [Internet].

[27] Salomone S, Drago F (2012). Effects of PPARγ ligands on vascular tone. Curr. Mol. Pharmacol. [Internet].

[28] Toporsian M, Gros R, Kabir MG, Vera S, Govindaraju K, Eidelman DH (2005). A role for endoglin in coupling eNOS activity and regulating vascular tone revealed in hereditary hemorrhagic telangiectasia. Circ. Res. [Internet].

[29] Albrecht EWJA, Stegeman CA, Heeringa P, Henning RH, van Goor H (2003). Protective role of endothelial nitric oxide synthase. J. Pathol. [Internet].

[30] Takada I, Kouzmenko AP, Kato S (2009). Wnt and PPARgamma signaling in osteoblastogenesis and adipogenesis. Nat. Rev. Rheumatol. [Internet].

[31] Lecarpentier Y, Claes V, Duthoit G, Hébert J-L (2014). Circadian rhythms, Wnt/beta-catenin pathway and PPAR alpha/gamma profiles in diseases with primary or secondary cardiac dysfunction. Front. Physiol. [Internet]..

[32] Dejana E (2010). The role of wnt signaling in physiological and pathological angiogenesis. Circ. Res. [Internet].

[33] He B, Barg RN, You L, Xu Z, Reguart N, Mikami I (2005). Wnt signaling in stem cells and non-small-cell lung cancer. Clin. Lung Cancer [Internet].

[34] Pongracz JE, Stockley RA (2006). Wnt signalling in lung development and diseases. Respir. Res. [Internet].

[35] Rao TP, Kühl M (2010). An updated overview on Wnt signaling pathways: a prelude for more. Circ. Res. [Internet].

[36] Stewart DJ (2014). Wnt signaling pathway in non-small cell lung cancer. J. Natl. Cancer Inst. [Internet].

[37] Bartis D, Csongei V, Weich A, Kiss E, Barko S, Kovacs T (2013). Down-regulation of canonical and up-regulation of non-canonical Wnt signalling in the carcinogenic process of squamous cell lung carcinoma. PLoS One [Internet].

[38] Cheng C, Yeh J, Fan T-P, Smith SK, Charnock-Jones DS (2008). Wnt5a-mediated non-canonical Wnt signalling regulates human endothelial cell proliferation and migration. Biochem. Biophys. Res. Commun. [Internet]..

[39] Nadra K, Quignodon L, Sardella C, Joye E, Mucciolo A, Chrast R (2010). PPARgamma in placental angiogenesis. Endocrinology [Internet].

[40] Geraghty RJ, Capes-Davis A, Davis JM, Downward J, Freshney RI, Knezevic I (2014). Guidelines for the use of cell lines in biomedical research. Br. J. Cancer [Internet]..

[41] Haq S, Michael A, Andreucci M, Bhattacharya K, Dotto P, Walters B (2003). Stabilization of beta-catenin by a Wnt-independent mechanism regulates cardiomyocyte growth. Proc. Natl. Acad. Sci. U. S. A. [Internet].

[42] Whiteside C, Wang H, Xia L, Munk S, Goldberg HJ, Fantus IG (2009). Rosiglitazone prevents high glucose-induced vascular endothelial growth factor and collagen IV expression in cultured mesangial cells. Exp. Diabetes Res. [Internet].

[43] Seargent JM, Yates EA, Gill JH (2004). GW9662, a potent antagonist of PPARgamma, inhibits growth of breast tumour cells and promotes the anticancer effects of the PPARgamma agonist rosiglitazone, independently of PPARgamma activation. Br. J. Pharmacol. [Internet]..

[44] Kovacs T, Csongei V, Feller D, Ernszt D, Smuk G, Sarosi V (2014). Alteration in the Wnt microenvironment directly regulates molecular events leading to pulmonary senescence. Aging Cell [Internet].

[45] Bovia F (2002). Efficient transduction of primary human B lymphocytes and nondividing myeloma B cells with HIV-1-derived lentiviral vectors. Blood [Internet].

[46] Schindelin J, Arganda-Carreras I, Frise E, Kaynig V, Longair M, Pietzsch T (2012). Fiji: an open-source platform for biological-image analysis. Nat. Methods [Internet].

[47] Chintalgattu V, Harris GS, Akula SM, Katwa LC (2007). PPAR-gamma agonists induce the expression of VEGF and its receptors in cultured cardiac myofibroblasts. Cardiovasc. Res. [Internet]..

[48] Karbiener M, Fischer C, Nowitsch S, Opriessnig P, Papak C, Ailhaud G (2009). microRNA miR-27b impairs human adipocyte differentiation and targets PPARgamma. Biochem. Biophys. Res. Commun. [Internet]..

[49] Jennewein C, von Knethen A, Schmid T, Brüne B (2010). MicroRNA-27b contributes to lipopolysaccharide-mediated peroxisome proliferator-activated receptor gamma (PPARgamma) mRNA destabilization. J. Biol. Chem. [Internet]..

[50] Veliceasa D, Biyashev D, Qin G, Misener S, Mackie AR, Kishore R (2015). Therapeutic manipulation of angiogenesis with miR-27b. Vasc. Cell [Internet].

[51] Hannafon BN, Carpenter KJ, Berry WL, Janknecht R, Dooley WC, Ding W-Q (2015). Exosome-mediated microRNA signaling from breast cancer cells is altered by the anti-angiogenesis agent docosahexaenoic acid (DHA). Mol. Cancer [Internet]..

[52] Choi Y-C, Yoon S, Jeong Y, Yoon J, Baek K (2011). Regulation of vascular endothelial growth factor signaling by miR-200b. Mol. Cells [Internet]..

[53] Hoeben A, Landuyt B, Highley MS, Wildiers H, Van Oosterom AT, De Bruijn EA (2004). Vascular endothelial growth factor and angiogenesis. Pharmacol. Rev. [Internet]..

[54] Liu Z, Lebrin F, Maring JA, van den Driesche S, van der Brink S, van Dinther M (2014). ENDOGLIN is dispensable for vasculogenesis, but required for vascular endothelial growth factor-induced angiogenesis. PLoS One [Internet].

[55] Schmidinger M, Bellmunt J (2010). Plethora of agents, plethora of targets, plethora of side effects in metastatic renal cell carcinoma. Cancer Treat. Rev. [Internet].

[56] Kamba T, McDonald DM (2007). Mechanisms of adverse effects of anti-VEGF therapy for cancer. Br. J. Cancer [Internet].

[57] Reck M, Barlesi F, Crinò L, Henschke CI, Isla D, Stiebeler S (2012). Predicting and managing the risk of pulmonary haemorrhage in patients with NSCLC treated with bevacizumab: a consensus report from a panel of experts. Ann. Oncol. [Internet].

[58] Li X-Q, Yang X-L, Zhang G, Wu S-P, Deng X-B, Xiao S-J (2013). Nuclear β-catenin accumulation is associated with increased expression of Nanog protein and predicts poor prognosis of non-small cell lung cancer. J. Transl. Med. [Internet].

[59] Yao L, Sun B, Zhao X, Zhao X, Gu Q, Dong X (2014). Overexpression of Wnt5a promotes angiogenesis in NSCLC. Biomed Res. Int. [Internet].

[60] Biselli-Chicote PM, Oliveira ARCP, Pavarino EC, Goloni-Bertollo EM (2012). VEGF gene alternative splicing: pro- and anti-angiogenic isoforms in cancer. J. Cancer Res. Clin. Oncol. [Internet]..

[61] Lee A, Frischer J, Serur A, Huang J, Bae J-O, Kornfield ZN (2006). Inhibition of cyclooxygenase-2 disrupts tumor vascular mural cell recruitment and survival signaling. Cancer Res. [Internet]..

[62] Bishop-Bailey D, Swales KE, Bishop-Bailey D, Swales KE (2008). The Role of PPARs in the Endothelium: Implications for Cancer Therapy. PPAR Res. [Internet].

[63] McArthur K, Feng B, Wu Y, Chen S, Chakrabarti S (2011). MicroRNA-200b regulates vascular endothelial growth factor-mediated alterations in diabetic retinopathy. Diabetes [Internet]..

[64] Thulasingam S, Massilamany C, Gangaplara A, Dai H, Yarbaeva S, Subramaniam S (2011). miR-27b*, an oxidative stress-responsive microRNA modulates nuclear factor-kB pathway in RAW 264.7 cells. Mol. Cell. Biochem. [Internet].

[65] Graham JR, Williams CMM, Yang Z (2014). MicroRNA-27b targets gremlin 1 to modulate fibrotic responses in pulmonary cells. J. Cell. Biochem. [Internet].

[66] Tabruyn SP, Griffioen AW (2007). A new role for NF-κB in angiogenesis inhibition. Cell Death Differ. [Internet].

[67] Mitola S, Ravelli C, Moroni E, Salvi V, Leali D, Ballmer-Hofer K (2010). Gremlin is a novel agonist of the major proangiogenic receptor VEGFR2. Blood [Internet].

