# Supplementary material for: Increased Wnt5a in squamous cell lung carcinoma inhibits endothelial cell motility
Source: BMC Cancer. 2016 Nov 23;16:915. doi: 10.1186/s12885-016-2943-4 (PMC5120464; doi:10.1186/s12885-016-2943-4)
Supplement: Additional file 1: — Table S1. Characteristics of patients. The table contains number, histological types, sex, age and pathological TNM status of all patients. (DOCX 14 kb) [file 12885_2016_2943_MOESM1_ESM.docx]

**Table S1**

| **Histologic type** | **Adenocarcinoma**  **(n=23)** | **Squamous cell carcinoma**  **(n=16)** |
| --- | --- | --- |
| **Sex (n)**  Female  Male | 8  11 | 3  12 |
| **Age (yr)**  Median  Range | 59  41-73 | 59.5  54-77 |
| **Pathological TNM stage**  T1N0  T1N1  T1N2  T2N0  T2N1  T2N2  T3N0  T3N1  T3N2  T4N0 | 4  -  3  4  5  2  1  1  3  - | 2  1  1  8  1  2  -  2  -  1 |
