# Supplementary figures and images for: Increased Wnt5a in squamous cell lung carcinoma inhibits endothelial cell motility
Source: BMC Cancer. 2016 Nov 23;16:915. doi: 10.1186/s12885-016-2943-4 (PMC5120464; doi:10.1186/s12885-016-2943-4)

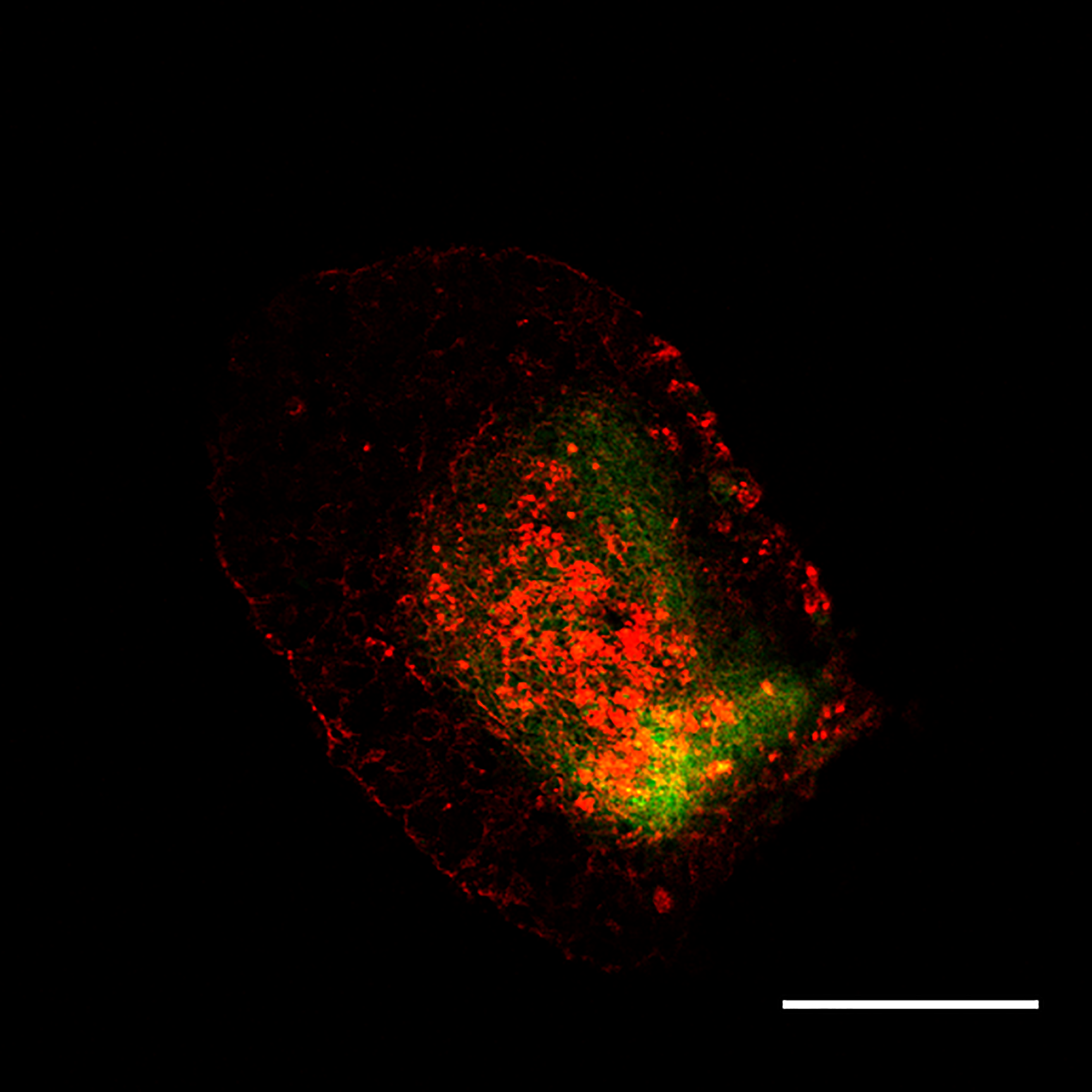

Supplement: Additional file 2: — Figure S1. 3D human lung tissue aggregates to study angiogenesis. To investigate the molecular background of angiogenesis, a three dimensional human lung tissue model was set up using three characteristic cell types of the lung. Namely, primary normal small airway epithelial cells (SAEC), normal human lung fibroblast (NHLF) or VEGF-A-GFP overexpressing human fibroblast (F11) and human microvascular endothelial cells lung subtype (HMVEC-L). GFP aided the visualization of VEGF-A overexpressing fibroblasts in the core of the lung model. Actin was labeled by Alexa Fluor 568 conjugated phalloidin (Thermo Fisher Scientific, Waltham, USA) to detect all cells in the tissue. Scale bar, 200 μm. (TIF 1897 kb) [file 12885_2016_2943_MOESM2_ESM.tif]

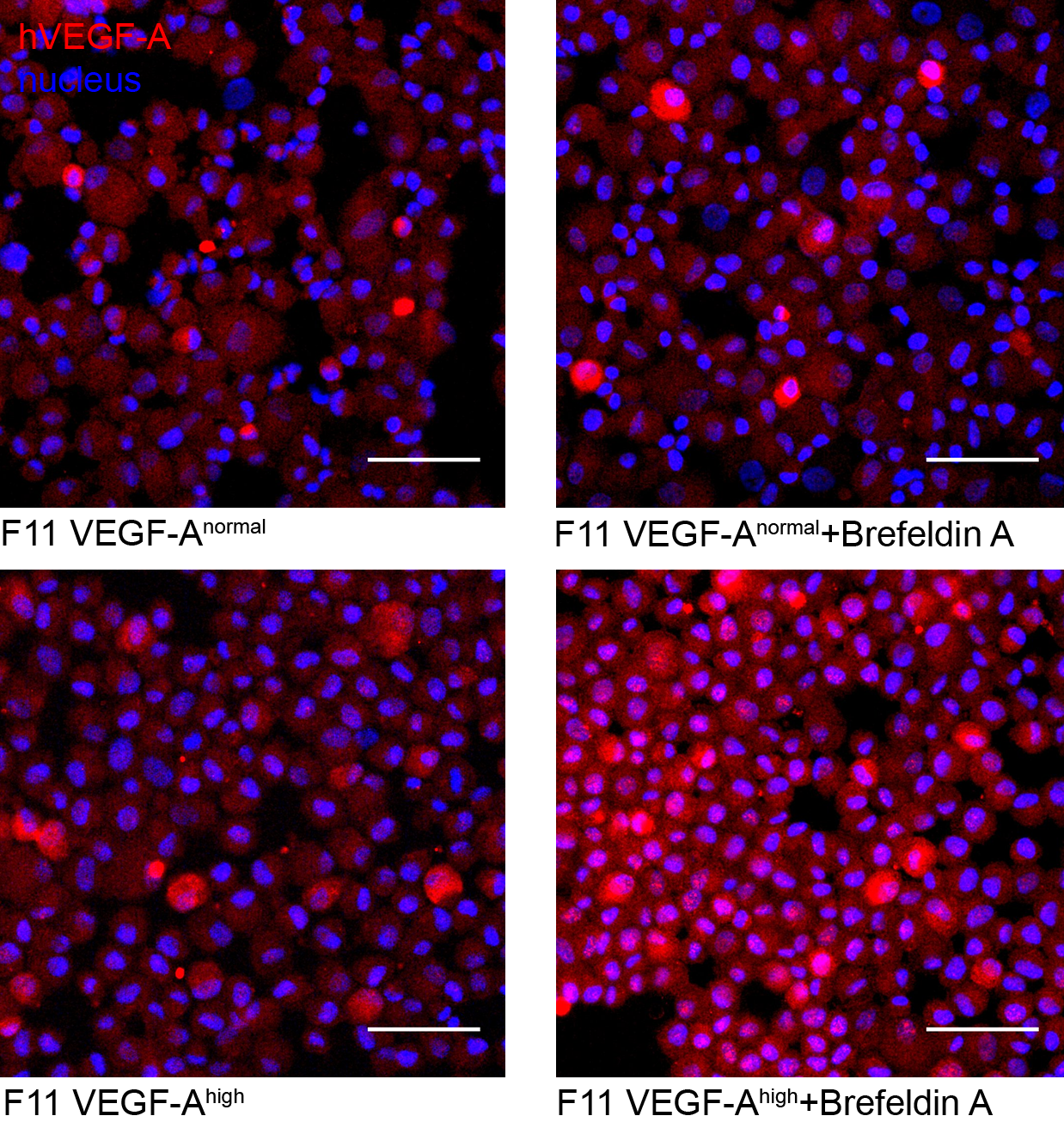

Supplement: Additional file 4: — Figure S3. Overexpression of VEGF-A in F11 cell line. Human VEGF-A-IRES-GFP was cloned into human F11 cell line. Overexpression of hVEGF-A protein was analyzed by fluorescent staining using anti-human VEGF-A as primary antibody. The secondary antibody was Northern Light anti-mouse NL-557. As VEGF-A is a secreted, soluble factor, cells were treated with brefeldin-A at 10 μg/ml for 4 h to inhibit protein transport. Accumulation of the protein was detected after brefeldin-A treatment. Images were captured using Olympus IXB1 fluorescence microscopy equipped with CCD camera. Scale bars, 50 μm. (TIF 3030 kb) [file 12885_2016_2943_MOESM4_ESM.tif]

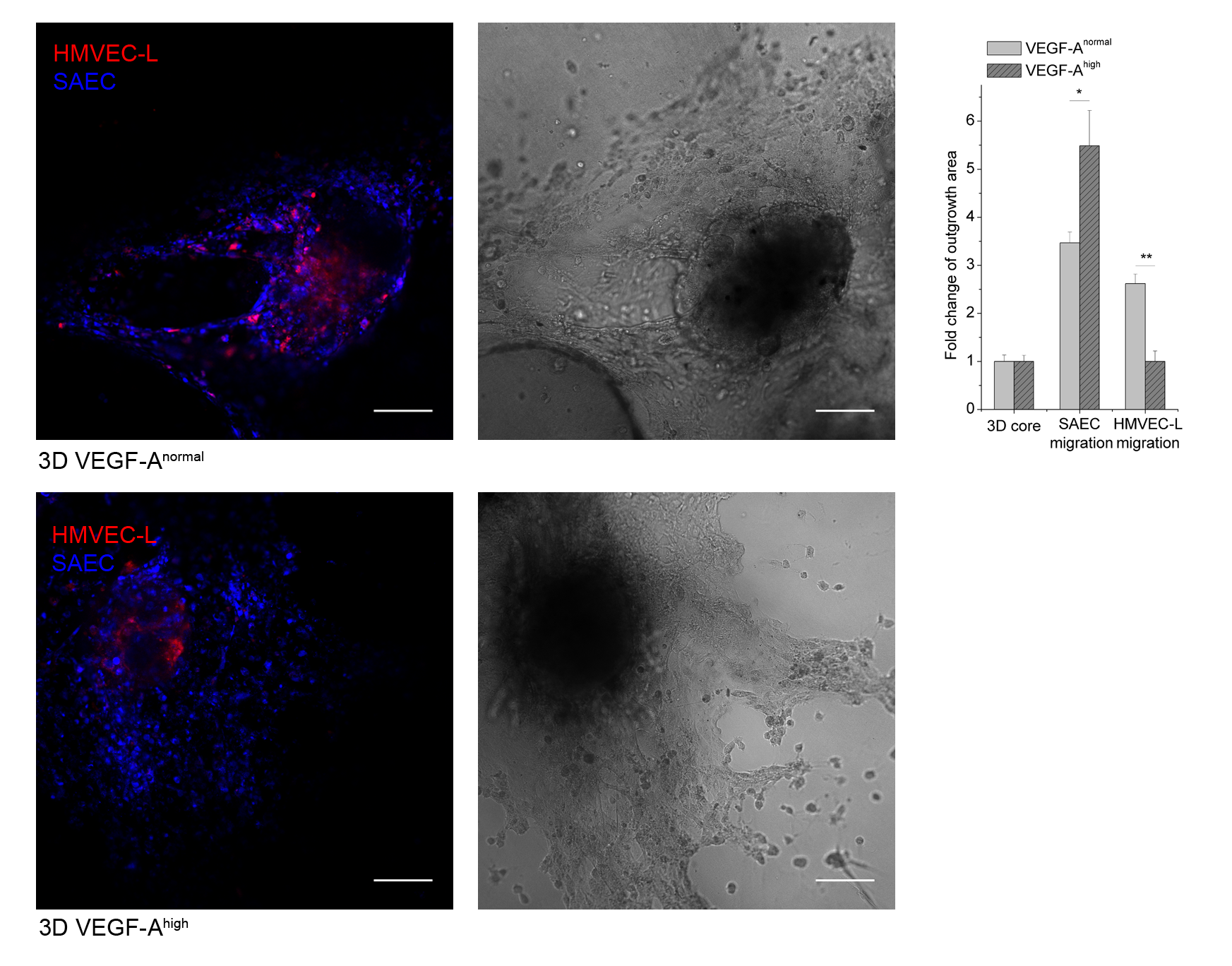

Supplement: Additional file 5 — Figure S4. Area of sprout outgrowth from 3D lung aggregates in normal and high VEGF-A microenvironment. Endothelial cells are evenly distributed in VEGF-Anormal microenvironment, while in VEGF-Ahigh microenvironment the endothelial cells remained close to the VEGF-A source. Data are representation of three independent experiments. Independent samples t-test. Scale bars, 200 μm. (TIF 1597 kb) [file 12885_2016_2943_MOESM5_ESM.tif]

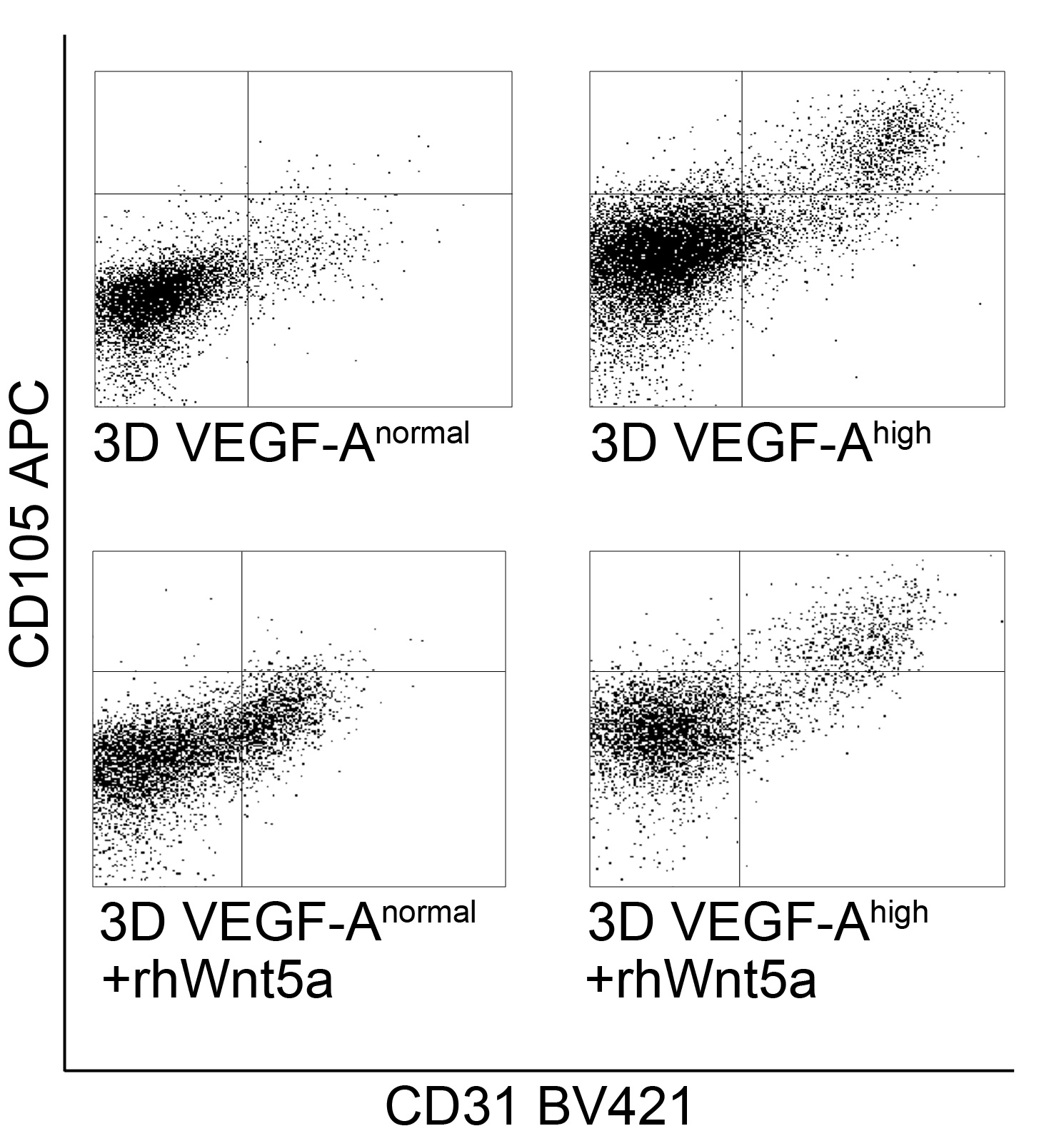

Supplement: Additional file 6: — Figure S5. Flow cytometric analysis of VEGF-A overexpressing 3D lung aggregates in the absence or presence of rhWnt5a. Representative dot plots of flow cytometric analysis of five independent experiments. 3D tissue cultures were created using normal VEGF-A and VEGF-Ahigh fibroblasts, respectively. Then the cultures were incubated in the presence or absence of rhWnt5a, dissociated and stained with anti-CD105 and anti-CD31 antibodies and analyzed in a flow cytometer. (TIF 469 kb) [file 12885_2016_2943_MOESM6_ESM.tif]

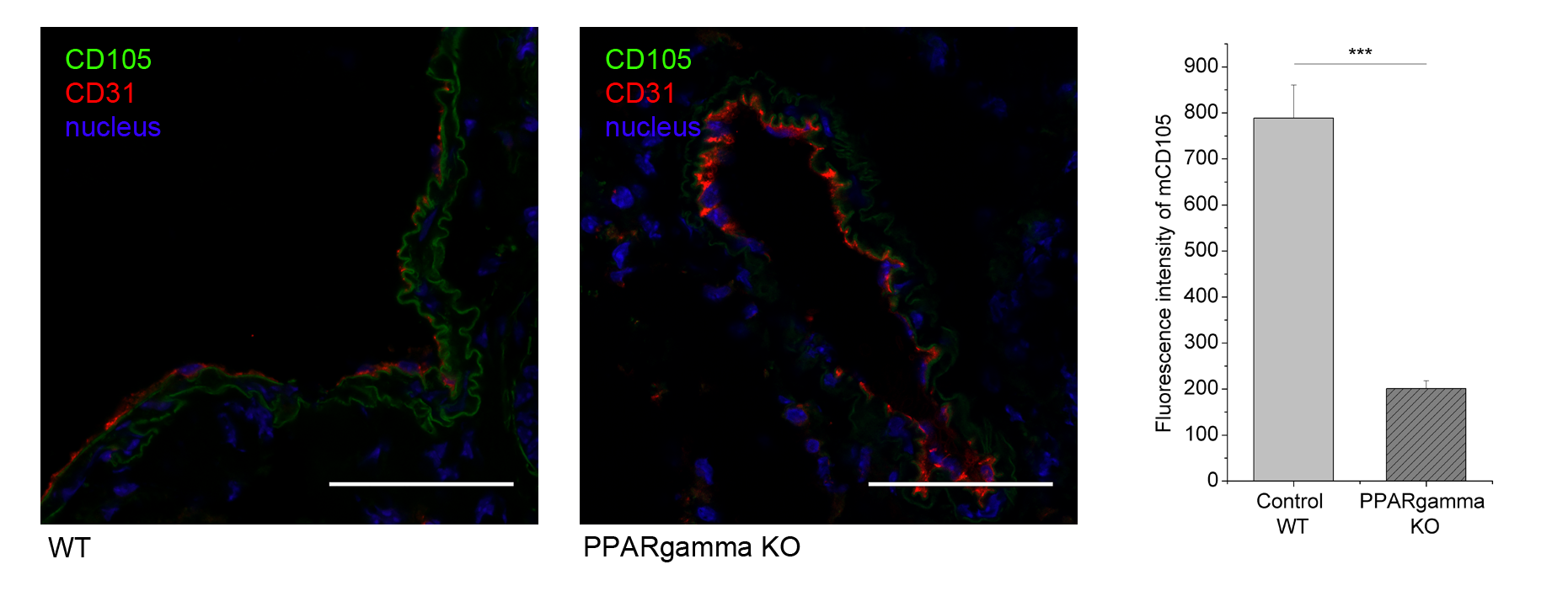

Supplement: Additional file 7: — Figure S6. Endoglin (CD105) expression of CD31+ endothelial cells in the lung of WT and PPARgamma KO mice. Immunfluorescence staining of wild type and PPARgamma KO mice showed decreased level of CD105 protein expression in PPARgamma KO lung tissues. Intensity data are representation of three individual experiments. Scale bars 50 μm. Staining intensity was quantified and presented in a bar chart. Error bars, SEM. Independent samples t-test, n = 3. P < 0.05 was considered as significant, P < 0.001. (TIF 3853 kb) [file 12885_2016_2943_MOESM7_ESM.tif]

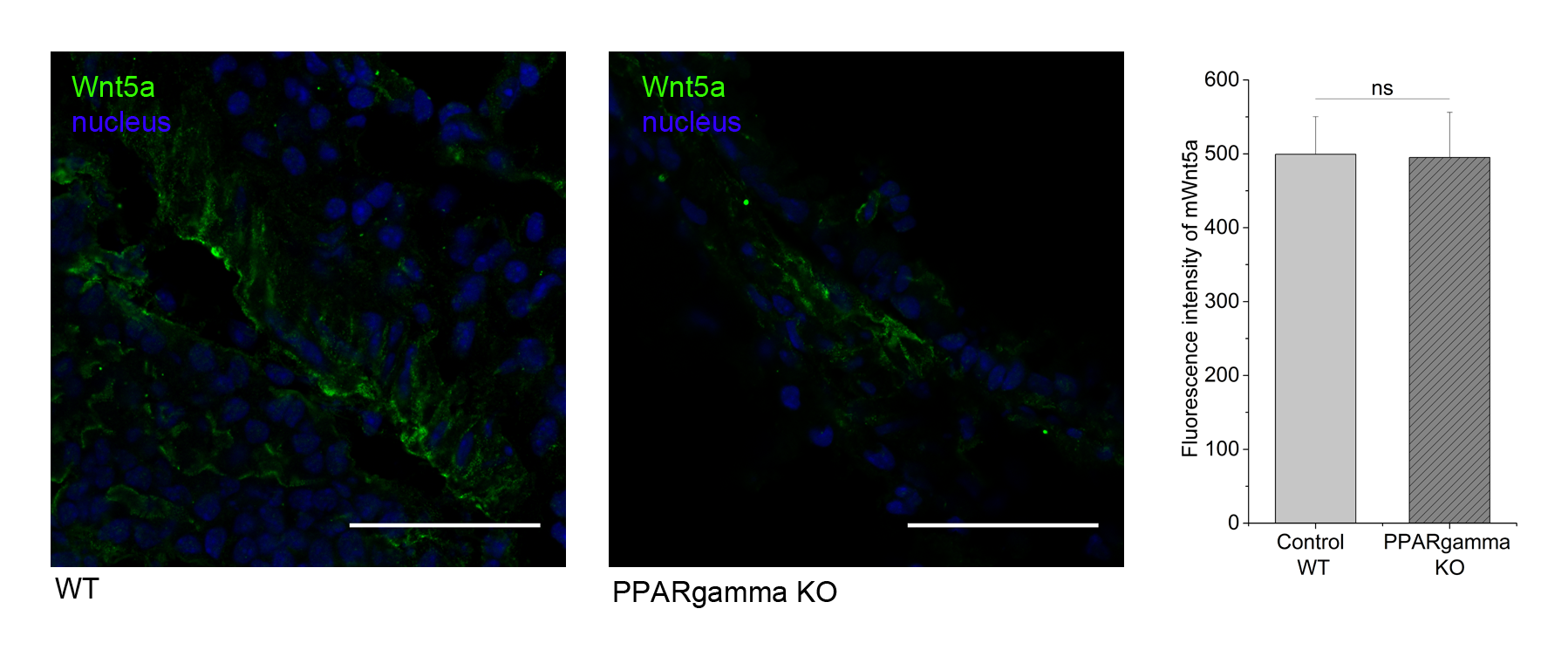

Supplement: Additional file 8: — Figure S7. Wnt5a expression in the lung of WT and PPARgamma KO mice. No differences were detected in Wnt5a protein expression in wild type and PPARgamma KO mice. (Scale bars 50 μm). The stainings of lung tissue sections are representatives of three individual experiments. Fluorescence intensity was quantified and presented in a bar chart. Error bars, SEM. Independent samples t-test, n = 3. P < 0.05 was considered as significant, ns: not significant. (TIF 769 kb) [file 12885_2016_2943_MOESM8_ESM.tif]
